# Overexpression of Class III β-tubulin, Sox2, and nuclear Survivin is predictive of taxane resistance in patients with stage III ovarian epithelial cancer

**DOI:** 10.1186/s12885-015-1553-x

**Published:** 2015-07-23

**Authors:** Jintong Du, Bei Li, Yingli Fang, Yanguo Liu, Yang Wang, Jisheng Li, Wen Zhou, Xiuwen Wang

**Affiliations:** 1 Department of Chemotherapy, Qilu Hospital of Shandong University, No. 107 Wenhuaxi road, Ji’nan, Shandong 250012 China; 2 Shandong Cancer Hospital, Shandong Academy of Medical Science, Ji’nan, Shandong 250012 China

**Keywords:** Ovarian cancer, Class III β-tubulin, Sox2, Survivin, Taxane, Resistance, Survival

## Abstract

**Background:**

Class III β-tubulin, Sox2, and Survivin play important roles in tumor survival and proliferation. However, the association of these three factors with clinicopathological characteristics, chemoresistance, and survival in patients with ovarian cancer remains controversial.

**Methods:**

We investigated the predictive value and correlation among the expression levels of Class III β-tubulin, Sox2, and Survivin in 110 patients with stage III ovarian epithelial cancer, including 58 patients who received taxane-based chemotherapy and 52 patients who received non-taxane-based chemotherapy. Expression of these three factors was immunohistochemically examined in 110 ovarian tumor tissues obtained from patients before chemotherapy.

**Results:**

The positive expression rates for Class III β-tubulin, Sox2, and Survivin in ovarian tumor tissues were 59.09 %, 61.82 % and 52.73 %, respectively. The expression of nuclear Survivin and Class III β-tubulin was consistent with that of Sox2 (*p* = 0.005 and 0.020, respectively). Positive expression of Class III β-tubulin, Sox2, and nuclear Survivin was significantly associated with chemoresistance to taxane-based chemotherapy (*p* = 0.006, 0.007, and 0.009, respectively), but not to non-taxane-based chemotherapy. Additionally, overexpression of Class III β-tubulin, Sox2, and nuclear Survivin predicted poor progression-free survival in patients receiving taxane-based chemotherapy (*p* = 0.032, 0.005, and 0.004, respectively).

**Conclusions:**

These findings suggest that overexpression of Class III β-tubulin, Sox2, and nuclear Survivin might be predictive of taxane resistance and poor progression-free survival in patients with stage III ovarian epithelial cancer. Expression of these three factors may show positive correlations in these patients.

**Electronic supplementary material:**

The online version of this article (doi:10.1186/s12885-015-1553-x) contains supplementary material, which is available to authorized users.

## Background

Ovarian cancer is regarded as the most lethal gynecologic malignancy and ranks as the seventh leading cause of cancer death among women [1]. The majority of patients with ovarian cancer are diagnosed at an advanced stage. Patients treated with standard therapies such as cytoreductive surgery and chemotherapy often experience tumor progression and poor survival, which may be due to intrinsic or acquired chemoresistance. In the past few decades, much research has been performed to identify predictive markers for ovarian cancer.

Class III β-tubulin has been linked to taxane resistance through a reduced microtubule polymerization rate. In 1997, Maria Kavallaris’s group first reported altered expression of specific β-tubulin genes in taxol resistant ovarian tumors and proposed that the class III and IVa isotypes of β-tubulin may play a role in clinical resistance to paclitaxel [2]. Several recent studies also suggested that the overexpression of Class III β-tubulin was related to paclitaxel resistance in ovarian cancer cell lines [3, 4].

The transcription factor sex-determining region Y box2 (Sox2), located on chromosome 3q26.3-q27 [5], plays a pivotal role in maintaining self-renewal and pluripotency of cancer stem cells (CSCs) and regulating tumor cell survival [6]. Persistence of CSCs could be detected in mouse ovarian cancer cells after paclitaxel/carboplatin chemotherapy, and may lead to tumor recurrence [7]. A study using human ovarian cancer cell lines also suggested that the expression of Sox2 might account for cellular resistance to paclitaxel, cisplatin, and carboplatin [8].

Survivin, the smallest member of the inhibitor of apoptosis protein family, prevents programmed cell death [9]. Similar to Class III β-tubulin, Survivin also interacts with microtubules of the mitotic spindle to oppose the action of taxane, which blocks cell division by stabilizing microtubules in the G2/M phase [10]. One *in vitro* study demonstrated that silencing of Survivin could increase the sensitivity of ovarian cancer cells to paclitaxel, but not to cisplatin [3, 11].

Interestingly, knockdown of the *Sox2* gene inhibited androgen-independent prostate cancer cell proliferation and induced apoptosis through downregulation of the *Survivin* gene [12]. Similar results were observed in human non-small-cell lung cancer cells [6]. Furthermore, overexpression of Sox2, induced by upregulation of Survivin, could maintain the survival and homoeostasis of neural stem cells [13]. Additionally, several researchers have reported that the transcriptions of Class III β-tubulin, Sox2, and Survivin could be induced by a common factor—hypoxia inducible factor—a key intermediate factor in the evolution of cancer [14–17]. These studies underscore the necessity of exploring the correlation between Sox2, its potential target gene Survivin, and Class III β-tubulin in ovarian cancer.

Although efforts have been made to delineate the relationship between these three factors and ovarian cancer [3, 4, 18–28] or other carcinomas [29–36], consensus conclusions still could not be reached because of contradictory results. The main reason for such discrepancy is probably the fact that most of these studies incorporated patients with some heterogeneity with respect to (1) clinical stage, (2) surgery patterns, (3) post-operative chemotherapy, (4) receipt of neoadjuvant chemotherapy, (5) evaluated index, (6) mRNA and/or protein level. Any of these factors could account for the unreliable or inconsistent results. Moreover, the correlations among Sox2, Survivin, and Class III β-tubulin have not been investigated among ovarian cancer. In addition, there are not enough data on the prognostic value of these three factors specifically in Chinese patients.

We performed a retrospective study of patients with stage III ovarian epithelial cancer (SOEC) who were treated with taxane-based or non-taxane-based chemotherapies. We investigated the correlations among Class III β-tubulin, Sox2, and Survivin, and the relationship between expression of these three factors and clinicopathologic characteristics, chemoresistance, and survival.

## Methods

### Patients

The study was performed using ovarian tumor tissues obtained from 110 consecutive patients with ovarian epithelial cancer in the Tumor Center and Department of Gynecology, Qilu Hospital of Shandong University, between 2000 and 2012. The Ethics Committee of Qilu Hospital approved this protocol, and all patients gave written informed consent. All patients met the following eligibility criteria: (1) classified stage III disease according to International Federation of Gynecologists and Obstetricians and the World Health Organization; (2) received at least two cycles of taxane-based or non-taxane-based chemotherapy beginning 2–3 weeks after primary cytoreductive surgery; (3) did not receive neoadjuvant chemotherapy before primary cytoreductive surgery. Administration of alternative chemotherapy regimens was mainly based on National Comprehensive Cancer Network guidelines, with consideration of anaphylaxis to taxane, the patients’ economic factors, and systematic practice variations in different treatment areas. The clinicopathologic characteristics of all patients are listed in Table 1.

**Table 1:** Clinicopathologic characteristics of 110 SOEC patients

Characteristics	Total no.	Taxane-based group	Non-taxane-based group	*P*
Total no.	110	58	52	
Median age (range)	54 (21–76)	54 (30–76)	54 (21–73)	
Age (years)				0.437
<65	94	51	43	
≥65	16	7	9	
Histotype				0.095
Serous	84	48	36	
Others^a^	26	10	16	
Grade				0.120
G1-2	38	21	17	
G3	72	37	35	
Ascites (mL)				0.193
<1000	67	32	35	
≥1000	43	26	17	
Residual tumor at surgery (cm)				0.513
<1	43	21	22	
≥1	67	37	30	
Median cycle of chemotherapy (range)	6 (2–8)	6 (2–8)	6 (2–8)	

^**a**^Ovarian endometrioid carcinoma, 11 cases; ovarian mucous carcinoma, 4 cases; ovarian clear cell carcinoma, 1case; ovarian malignant mixed müllerian tumor, 1 case; ovarian mixed adenocarcinoma, 9 cases

All patients received a median of six cycles of chemotherapy using a 21-day cycle after the primary cytoreductive surgery: 58 patients were treated with taxane-based chemotherapy (defined as the taxane-based group), i.e., PT (135–175 mg/m^2^ paclitaxel or 75 mg/m^2^ docetaxel on day 1 plus carboplatin dosed with an area under the curve of 4–6 or 75 mg/m^2^ cisplatin on day 2), and 52 patients were treated with non-taxane-based chemotherapy (defined as the non-taxane-based group), i.e., PC (carboplatin dosed with an area under the curve of 4–6 or 75 mg/m^2^ cisplatin on day 1 plus 750 mg/m^2^ cyclophosphamide on day 1), PAC (50 mg/m^2^ cisplatin on day 1 plus 550 mg/m^2^ cyclophosphamide on day 1 plus 35 mg/m^2^ doxorubicin on day 1), or TC (75 mg/m^2^ cisplatin on day 1 plus 0.75 mg/m^2^ topotecan on days 1–5).

Response to chemotherapy was evaluated according to the Response Evaluation Criteria in Solid Tumor (RECIST, version 1.1), which includes complete response (CR), partial response (PR), stable disease (SD), and progression of disease (PD). Progression-free survival (PFS) was calculated as the time from the start of chemotherapy to tumor progression or the last follow-up. Overall survival (OS) was calculated as the time from the beginning of chemotherapy to death or the last follow-up [33]. In this study, the median follow-up time was 35 months (range, 7–154 months).

### Immunohistochemistry

Paraffin-embedded sections of ovarian cancer tissue were obtained during primary cytoreductive surgery for all cases. Slides were deparaffinized, rehydrated, subjected to epitope retrieval, and treated with H_2_O_2_ to block endogenous peroxidase activity. The slides were incubated with normal rabbit or goat serum, followed by incubation with a polyclonal goat anti-Sox2 antibody (AF2018, RD Systems, USA), a monoclonal rabbit anti-Survivin antibody (ab76424, Abcam, USA), and a monoclonal rabbit anti-Class III β-tubulin antibody (ab52623, Abcam, USA), overnight at 4 °C. Detection was performed using the Streptavidin/Peroxidase kit, Polymer HRP Detection system and DAB kit. Slides were counterstained with hematoxylin and dehydrated with alcohol and xylene. Positive controls were provided by slides taken from glioma tissues for Sox2 and Class III β-tubulin, and from colon cancer tissue for Survivin. Negative controls were provided by replacing the primary antibody with phosphate-buffered saline.

The intensity of staining and the percentage of stained cells were evaluated under a light microscope by three independent pathologists without knowledge of clinical data. The intensity of staining was evaluated as follows: 1 = weak staining, 2 = moderate staining, 3 = strong staining. The percentage of stained cells was categorized as follows: 0 = 0 % positive cells, 1 = 1–10 % positive cells, 2 = 11–35 % positive cells, 3 = 36–65 % positive cells, 4 = 66–100 % positive cells. The total score of stained cells was calculated by the sum of above two scores, where grade 0 = 0, grade 1 = 2–3, grade 2 = 4, grade 3 = 5, grade 4 = 6–7. Tumor tissues with grade 2–4 were defined as positive expression and those with grade 0–1 as negative expression.

### Statistical analysis

Associations between expression of the three factors (Class III β-tubulin, Sox2, and Survivin) and clinicopathologic characteristics or response to chemotherapy were tested using *χ*^2^ test or Fisher’s exact test, as appropriate. Survival curves were estimated using the Kaplan–Meier method, and differences in PFS and OS of two subgroups were evaluated using the log-rank test. Cox regression was used for univariate analysis and multivariate analysis to evaluate the prognostic value of the three factors for survival. Only variables with *P* < 0.10 in the univariate analysis were included in the multivariate model. All statistical analyses were carried out using SPSS 13.0.

## Results

### Expression of Class III β-tubulin, Sox2, and Survivin

**Fig. 1:**
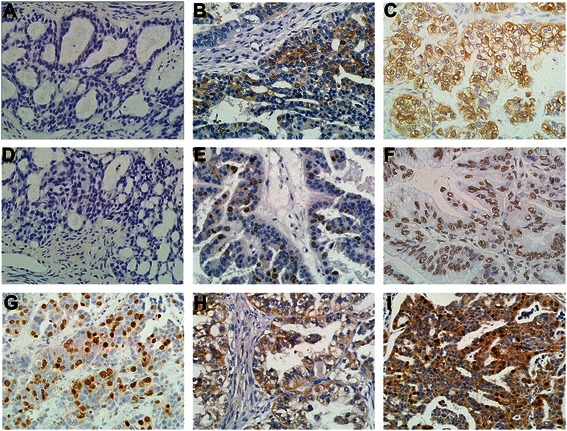
Representative pictures by immunochemistry for Class III β-tubulin, Sox2, and Survivin in 110 SOEC patients. Magnification 400×. Class III β-tubulin expression: (**a**) negative expression in serous carcinoma, (**b**) low expression in serous carcinoma, (**c**) high expression in clear carcinoma. Sox2 expression: (**d**) negative expression in serous carcinoma, (**e**) low expression in serous carcinoma, (**f**) high expression in mucous carcinoma. Survivin expression: (**g**) only nuclear expression in serous carcinoma, (**h**) only cytoplasmic expression in serous carcinoma, (**i**) both nuclear and cytoplasmic expression in serous carcinoma

Among the 110 cases, 65 (59.09 %), 68 (61.82 %), and 58 (52.73 %) were positive for expression of Class III β-tubulin, Sox2, and Survivin, respectively. As shown in Fig. 1, the expression of Class III β-tubulin and Sox2 was detected in both the cytoplasm and nucleus in all cases. The expression of Survivin differed among cases: 35 cases (60.34 %) showed expression only in the nucleus, 9 (15.52 %) only in the cytoplasm, and 14 (24.14 %) in both the nucleus and cytoplasm.

It should be mentioned that the expression of nuclear Survivin and Class III β-tubulin was significantly linearly correlated with that of Sox2 (*p* = 0.005 and 0.020), as shown in Table 2. However, cytoplasmic Survivin did not show a similar association with Sox2 (*p* = 0.307).

**Table 2:** Correlations among Class III β-tubulin, Sox2, and Survivin in 110 SOEC patients

	Total no.	Sox2 positive	Sox2 negative	*P* ^a^
Nuclear Survivin				0.005*
Total no.	101	62	39	
Positive	49	37	12	
Negative	52	25	27	
Cytoplasmic Survivin				0.307
Total no.	75	39	36	
Positive	23	14	9	
Negative	52	25	27	
Class III β-tubulin				0.020*
Total no.	110	68	42	
Positive	65	46	19	
Negative	45	22	23	

^a^Calculated by *χ*^2^ test

^*^P < 0.05

### Associations between expression of Class III β-tubulin, Sox2, and Survivin and clinicopathologic characteristics and response to chemotherapy

As shown in Table 3, expression of Class III β-tubulin, Sox2, and Survivin was not significantly correlated with age, histotype, grade, ascites, or residual tumor (*p* > 0.05).

**Table 3:** Associations between expression of Class III β-tubulin, Sox2, and Survivin and clinicopathologic characteristics in 110 SOEC patients

Characteristics	Class III β-tubulin positive/negative	*P* ^a^	Sox2 positive/negative	*P* ^a^	Only nuclear Survivin positive/negative	*P* ^a^	Only cytoplasmic Survivin positive/negative	*P* ^a^
Total no.	65/45		68/42		35/52		9/52	
Age (years)		0.424		0.620		0.707		1.000
<65	57/37		59/35		30/43		8/43	
≥65	8/8		9/7		5/9		1/9	
Histotype		0.097		0.176		0.733		0.224
Serous	46/38		49/35		28/40		5/40	
Others	19/7		19/7		7/12		4/12	
Grade		0.101		0.202		0.396		1.000
G1-2	19/20		47/24		11/21		3/21	
G3	46/25		10/5		24/31		6/31	
Ascites (mL)		0.814		0.568		0.371		0.725
<1000	39/28		40/27		18/30		6/30	
≥1000	26/17		28/15		17/19		3/22	
Residual tumor (cm)		0.575		0.299		0.305		0.279
<1	24/19		24/19		11/22		6/22	
≥1	41/26		44/23		24/30		3/30	

^a^Calculated by *χ*^2^ test, and Fisher’s exact test as appropriate

The relationships between these three factors and response to chemotherapy are shown in Table 4. In the taxane-based group, positive expression of Class III β-tubulin, Sox2, and only nuclear Survivin was significantly associated with disease progression (*p* = 0.006, 0.007, and 0.009, respectively). However, in the non-taxane-based group, no significant associations between expression of these three factors and response to chemotherapy was demonstrated (*p* > 0.05).

**Table 4:** Associations between expression of Class III β-tubulin, Sox2, and Survivin and response to chemotherapy^a^ in 100 SOEC patients

	Taxane-based group				Non-taxane-based group			
	Total no.	PR	PD	*P* ^b^	Total no.	PR	PD	*P* ^b^
Class III β-tubulin				0.006*				0.145
Total no.	57	34	23		43	33	10	
Positive	34	15	19		24	16	8	
Negative	23	19	4		19	17	2	
Sox2				0.007*				0.480
Total no.	57	34	23		43	33	10	
Positive	40	19	21		25	18	7	
Negative	17	15	2		18	15	3	
Only nuclear Survivin				0.009*				0.390
Total no.	41	24	17		37	30	7	
Positive	19	7	12		14	10	4	
Negative	22	17	5		23	20	3	
Only cytoplasmic Survivin				0.580				0.495
Total no.	27	20	7		27	23	4	
Positive	5	3	2		4	3	1	
Negative	22	17	5		23	20	3	

^a^Response to chemotherapy in 100 SOEC patients includes partial response (PR) and progression of disease (PD). There were no patients with complete response (CR) or stable disease (SD)

^b^Calculated by *χ*^2^ test

*P < 0.05

### Survival analysis

**Fig. 2:**
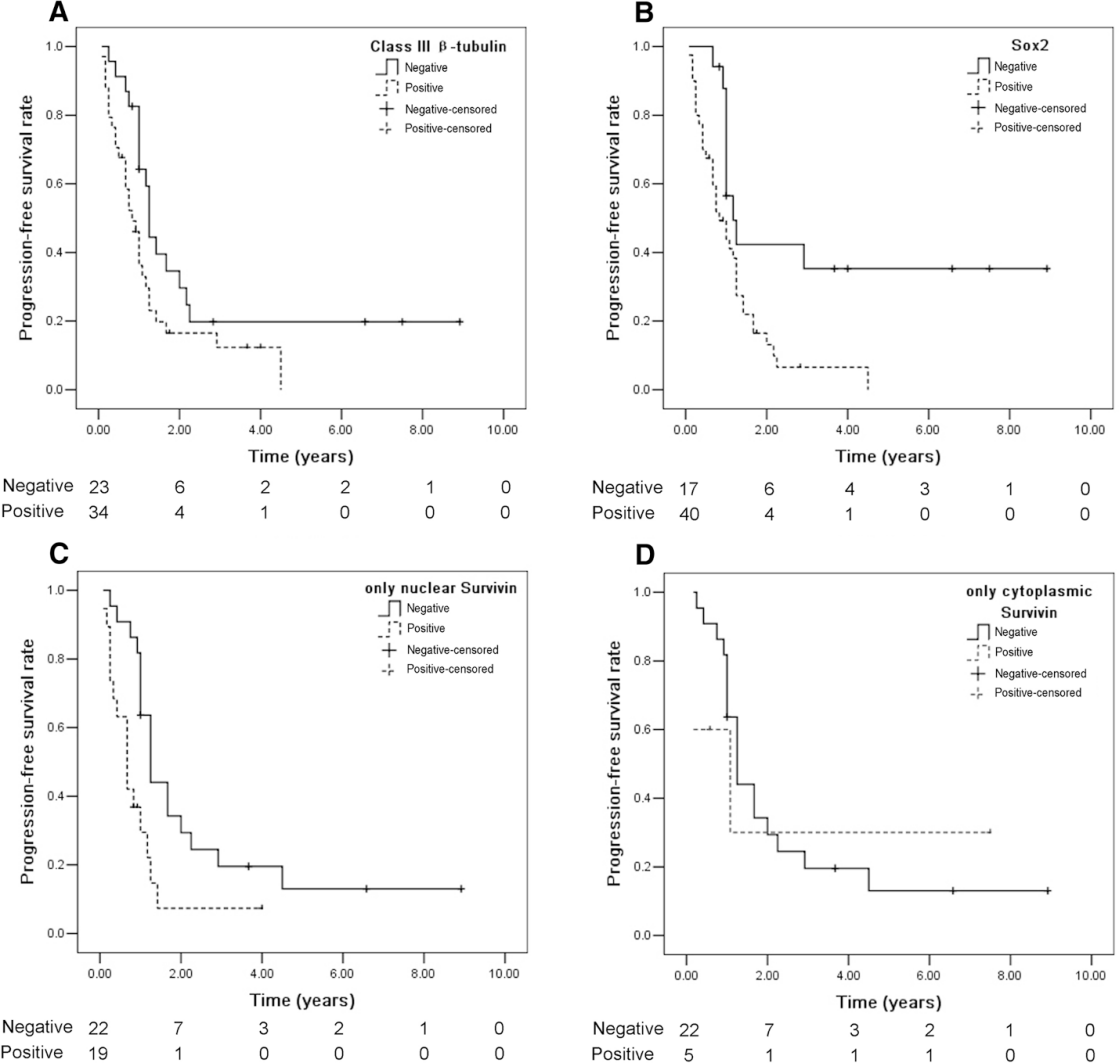
Kaplan-Meier analysis for progression-free survival (PFS) in 57 SOEC patients treated with taxane-based chemotherapy. Positive expression of Class III β-tubulin (**a**), Sox2 (**b**), and only nuclear Survivin (**c**) was associated with poor PFS (*p* = 0.032, 0.005, and 0.004, respectively). Expression of only cytoplasmic Survivin (**d**) was not related to PFS (*p* = 0.727). Number of patients at risk at the beginning of each year is shown below the horizontal axis

In the taxane-based group, progression-free survival (PFS) data were available for 57 patients (98.28 %), and the median PFS was 13 months. The Kaplan–Meier curves in Fig. 2 show that positive expression of Class III β-tubulin (A), Sox2 (B), and only nuclear Survivin (C) was associated with poor PFS (*p* = 0.032, 0.005 and 0.004, respectively). However, expression of only cytoplasmic Survivin (D) was not related to PFS (*p* = 0.727). The median PFS for patients with positive expression of Class III β-tubulin, Sox2, only nuclear Survivin and only cytoplasmic Survivin was 9.5, 9.5, 8, and 13 months, respectively; the median PFS for non-expressors of Class III β-tubulin, Sox2, only nuclear Survivin and only cytoplasmic Survivin was 15, 12, 15, and 15 months, respectively. The ratio of PFS for expressors and non-expressors of Class III β-tubulin, Sox2, only nuclear Survivin, and only cytoplasmic Survivin was 1.58, 1.58, 1.26, and 1.15, respectively.

The prognostic value of Class III β-tubulin, Sox2, and Survivin for PFS in the taxane-based group was also evaluated using univariate and multivariate analysis (Table 5). The univariate model indicated that residual tumor diameter >1 cm and positive expression of Class III β-tubulin, Sox2, and nuclear Survivin were associated with shorter PFS (*p* = 0.001, 0.042, 0.009, and 0.047, respectively). In multivariate analysis, residual tumor, Class III β-tubulin, and Sox2 remained as unfavorable independent prognostic variables for PFS (*p* = 0.002, 0.038, and 0.047, respectively).

**Table 5:** Univariate and multivariate Cox analysis of predictive factors for progression-free survival in 57 SOEC patients treated with taxane-based chemotherapy

Variables	Univariate			Multivariate		
	HR (95 % CI)	*χ* ^2^	*P*	HR (95 % CI)	*χ* ^2^	*P*
Age (years)	0.669 (0.282-1.586)	0.835	0.361	-	-	-
<65						
≥65						
Histotype	1.458 (0.720-2.950)	1.099	0.295	-	-	-
Serous						
Others						
Grade	1.244 (0.759-2.041)	0.750	0.386	-	-	-
G1-2						
G3						
Ascites (mL)	0.990 (0.552-1.774)	0.001	0.973	-	-	-
<1000						
>1000						
Residual tumor (cm)	3.333 (1.650-6.732)	11.266	0.001*	3.050 (1.499-6.205)	9.471	0.002*
<1						
≥1						
Class III β-tubulin	1.870 (1.022-3.423)	4.121	0.042*	1.915 (1.036-3.538)	4.301	0.038*
Positive						
Negative						
Sox2	2.623 (1.273-5.407)	6.829	0.009*	2.100 (1.009-4.373)	3.935	0.047*
Positive						
Negative						
Nuclear Survivin	1.838 (1.009-3.349)	3.959	0.047*	-	-	-
Positive						
Negative						
Cytoplasmic Survivin	0.958 (0.495-1.852)	0.016	0.898	-	-	-
Positive						
Negative						

*HR *hazard ratios, *CI* confidence interval

*P < 0.05; only variables with *P* < 0.10 in the univariate analysis were included in the multivariate model

In the non-taxane-based group, PFS data were available for 41 patients (78.85 %), and the median PFS was 20 months. Kaplan–Meier curves showed no significant associations between the three proteins and PFS [*p* = 0.408, 0.182, 0.386 and 0.965 for Class III β-tubulin (A), Sox2 (B), only nuclear Survivin (C), and only cytoplasmic Survivin (D), respectively]. The survival curves are shown in Additional file 1: Figure S1.

The overall survival (OS) of 98 patients (89.09 %) has been followed, and death occurred in 78 cases (79.59 %). The median OS for patients who received a taxane-based regimen was 55 months, and that for patients who received a non-taxane-based regimen was 58 months. No significant associations were observed between expression of the three proteins and OS of 98 patients using Kaplan–Meier analysis [*p* = 0.284, 0.138, 0.428, and 0.503 for Class III β-tubulin (A), Sox2 (B), the only nucear Survivin (C), and the only cytoplasmic Survivin (D), respectively]. The survival curves are shown in Additional file 2: Figure S2.

Potential prognostic factors for OS in all the 98 available patients were also evaluated by univariate and multivariate analysis (Table 6). Significant independent factors were response to chemotherapy, histotype, and residual tumor size (*p* < 0.0001, *p* = 0.002, and 0.010, respectively).

**Table 6:** Univariate and multivariate Cox analysis of predictive factors for overall survival in 98 SOEC patients

Variables	Univariate			Multivariate		
	HR (95 % CI)	*χ* ^2^	*P*	HR (95 % CI)	*χ* ^2^	*P*
Age (years)	1.176 (0.530-2.611)	0.159	0.690	-	-	-
<65						
≥65						
Histotype	1.390 (0.969-1.993)	3.198	0.074	2.015 (1.296-3.134)	9.680	0.002*
Serous						
Others						
Grade				-	-	-
G1-2	1.318 (0.862-2.016)	1.627	0.202			
G3						
Ascites (mL)	1.021 (0.576-1.811)	0.005	0.943	-	-	-
<1000						
>1000						
Residual tumor (cm)	3.814 (1.946-7.476)	15.204	<0.0001*	2.736 (1.271-5.890)	6.617	0.010*
<1						
≥1						
Response to chemotherapy	5.627 (3.078-10.289)	31.483	<0.0001*	5.318 (2.677-10.564)	22.770	<0.0001*
PR						
PD						
Class III β-tubulin	1.347 (0.776-2.339)	1.119	0.290	-	-	-
Positive						
Negative						
Sox2	1.537 (0.863-2.736)	2.128	0.145	-	-	-
Positive						
Negative						
Nuclear Survivin	1.399 (0.813-2.407)	1.472	0.225	-	-	-
Positive						
Negative						
Cytoplasmic Survivin	1.206 (0.643-2.261)	0.341	0.559	-	-	-
Positive						
Negative						

*HR *hazard ratios, *CI* confidence interval

*P < 0.05; only variables with *P* < 0.10 in the univariate analysis were included in the multivariate model

## Discussion

This retrospective study explored the predictive value of expression of Class III β-tubulin, Sox2, and Survivin, and correlations among these proteins in 110 SOEC patients who were treated with taxane- or non-taxane-based chemotherapy.

Our results demonstrated that positive expression of Class III β-tubulin was associated with disease progression in SOEC patients receiving taxane-based therapy, which is in line with previous studies [2, 21]. Furthermore, we found a correlation between overexpression of Class III β-tubulin and a shorter PFS in SOEC patients treated with taxane-based chemotherapy. Our results also suggested no significant relevance of Class III β-tubulin expression with response in patients who received non-taxane-based therapy. The Class III β-tubulin expression was not correlated with OS in the whole patient population. By defining disease progression under chemotherapy as chemoresistance [33, 34, 37], we found that overexpression of Class III β-tubulin was related to taxane resistance in SOEC patients. However, Ferrandina et al. reported that the Class III β-tubulin overexpression predicted a shorter OS and had no influence on taxane-based chemotherapy in patients with unresectable ovarian cancer [19]. Thus, Class III β-tubulin expression may present different biological characteristics in unresectable and resectable patients. Moreover, recent studies reported contradictory results regarding the predictive value of Class III β-tubulin for OS through immunohistochemistry or qRT-PCR tests in patients with stage I–IV ovarian cancer [4, 18, 20]. In general, it appears that different detection methods at the protein or mRNA level, as well as different clinical stages and chemotherapy regimens, may affect the identification of biomarkers.

The positive expression of Sox2 in the nucleus observed in the present study was in accordance with two previous studies [38, 39], but contradicted another study showing Sox2 expression in the cytoplasm [40]. It should be mentioned that we evaluated the predictive value of Sox2 in SOEC patients who received taxane- or non-taxane-based chemotherapy, which provides more detailed information than studies without subgroup analysis according to the type of chemotherapy [38, 39]. Our results showed that positive expression of Sox2 was correlated with chemoresistance and a shorter PFS in SOEC patients receiving taxane, whereas Sox2 expression had no significant relevance for response and PFS in patients receiving non-taxane-based chemotherapy. Thus, our study indicated a relationship between Sox2 and taxane-resistance in SOEC patients. However, Zhang et al. did not find an association between Sox2 and chemoresistance, probably due to heterogeneity of chemotherapies administered [38]. We also found that Sox2 was not a potential biomarker for OS, which was in accordance with data from Zhang’s group [38]. In contrast, Pham et al. reported that positive expression of Sox2 predicted a longer survival time in patients with stage II–IV and high-grade ovarian cancer [39]. These distinctions may be explained by differences in clinical stage, histotype, and chemotherapy regimens of patients enrolled in different studies.

Similar to several previous reports [41, 42], the present study indicates that expression of Survivin is predominantly nuclear rather than that of the cytoplasmic. Other studies have reported that expression of Survivin was nearly equivalent in nucleus and cytoplasm [26, 43], predominantly in the cytoplasm [27], or only in the cytoplasm [23], which may be due to differences in reagents, tissues and counting methods used. In contrast to these studies, we analyzed the expression of only nuclear Survivin and only cytoplasmic Survivin separately, to exclude any interference of the ratio of nuclear to cytoplasmic Survivin. Kaplan–Meier and univariate analyses showed that overexpression of nuclear Survivin was predictive of poor response to chemotherapy and short PFS in SOEC patients who received taxane-based chemotherapy, but not for those treated with non-taxane-based chemotherapy. However, the predictive value of nuclear Survivin for PFS was not significant in multivariate analysis, perhaps influenced by the sample size and interaction with other factors. In general, we proposed that positive expression of nuclear Survivin might be predictive for taxane-resistance in SOEC patients, expression of cytoplasmic Survivin has no significant predictive value. In most cases, it is the nuclear Survivin, rather than the cytoplasmic Survivin, that sustains cells’ pluripotency [44, 45], which may support the prognostic value of nuclear staining. Neither nuclear Survivin nor cytoplasmic Survivin was found to be associated with OS in patients. However, previous studies showed contradictory results when evaluating the predictive value of Survivin, which may be due to incorporation of different chemotherapies and clinical stages [3, 23, 24, 26, 28]. Interestingly, Vivas-Mejia et al. demonstrated that Survivin-2B was a prognostic biomarker in taxane-resistant ovarian epithelial cancer [25]. In light of these inconsistent results, the subcellular localization and splicing variants of Survivin should be further investigated.

Our data demonstrated that residual tumor size following primary cytoreductive surgery and histotype were independent predictive factors for PFS and OS in SOEC patients, which is in accordance with the features of favorable tumor biology [19, 46]. However, expression of Class III β-tubulin, Sox2, and Survivin showed no relationship with clinicopathologic characteristics and OS. The fact that some SOEC patients received other therapies when tumor progression was found after first-line chemotherapy may have some effect on patient characteristics and the expression of these proteins. Thus, we hypothesized that Class III β-tubulin, Sox2, and nuclear Survivin may be used to predict chemoresistance, but not the intrinsic tumor aggressiveness and OS.

Several *in vitro* studies have reported that Survivin is a downstream target of Sox2 [6, 12, 13]. Our results for nuclear Survivin and cytoplasmic Survivin at the protein level in ovarian cancer tissues indicated that nuclear Survivin expression was consist with Sox2 expression. Moreover, Class III β-tubulin expression also correlated with Sox2 expression, suggesting the need for further investigation into the clinical relevance of these associations.

The limitations of our study include the small sample size and its retrospective nature. The limited number of patients and missing information of follow-up in terms of PFS for patients who received non-taxane chemotherapies (21.15 %) may affect the significance of our results. A larger number of cases, and mRNA expression of these potential markers, should be investigated to confirm our findings.

## Conclusions

Overexpression of Class III β-tubulin, Sox2, and nuclear Survivin might predict taxane resistance and poor progression-free survival in patients with SOEC. Future prospective studies evaluating these markers in SOEC patients should be carried out to determine their clinical potential.

## Additional files

Additional file 1: Figure S1. Kaplan-Meier analysis for progression-free survival (PFS) in 41 SOEC patients treated with non-taxane-based chemotherapy. There were no significant associations between expression of the three proteins and PFS [*p* = 0.408, 0.182, 0.386 and 0.965 for Class III β-tubulin (A), Sox2 (B), only nuclear Survivin (C), and only cytoplasmic Survivin (D), respectively]. (JPEG 1010 kb)
Additional file 2: Figure S2. Kaplan-Meier analysis for overall survival (OS) in 98 SOEC patients. There were no significant associations between expression of the three proteins and OS [*p* = 0.284, 0.138, 0.428, and 0.503 for Class III β-tubulin (A), Sox2 (B), only nuclear Survivin (C), and only cytoplasmic Survivin (D), respectively]. (JPEG 1010 kb)

## Abbreviations

SOEC: Stage III ovarian epithelial cancer
CSCs: Cancer stem cells
CR: Complete response
PR: Partial response
SD: Stable disease
PD: Progression of disease
PFS: Progression-free survival
OS: Overall survival
