# Supplementary figures and images for: Overexpression of Class III β-tubulin, Sox2, and nuclear Survivin is predictive of taxane resistance in patients with stage III ovarian epithelial cancer
Source: BMC Cancer. 2015 Jul 23;15:536. doi: 10.1186/s12885-015-1553-x (PMC4511538; doi:10.1186/s12885-015-1553-x)

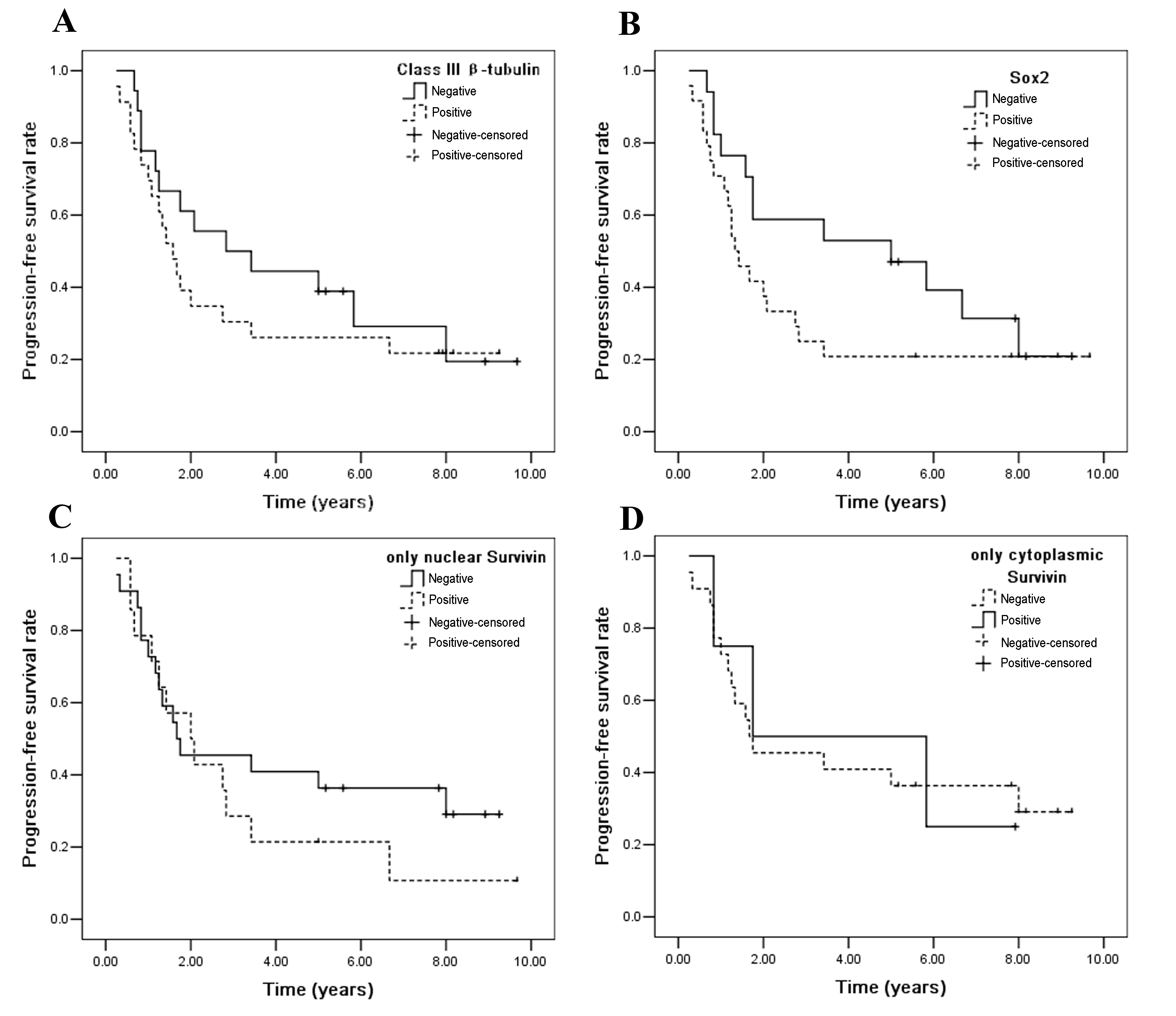

Supplement: Additional file 1: Figure S1. — Kaplan-Meier analysis for progression-free survival (PFS) in 41 SOEC patients treated with non-taxane-based chemotherapy. There were no significant associations between expression of the three proteins and PFS [p = 0.408, 0.182, 0.386 and 0.965 for Class III β-tubulin (A), Sox2 (B), only nuclear Survivin (C), and only cytoplasmic Survivin (D), respectively]. (JPEG 1010 kb) [file 12885_2015_1553_MOESM1_ESM.jpeg]

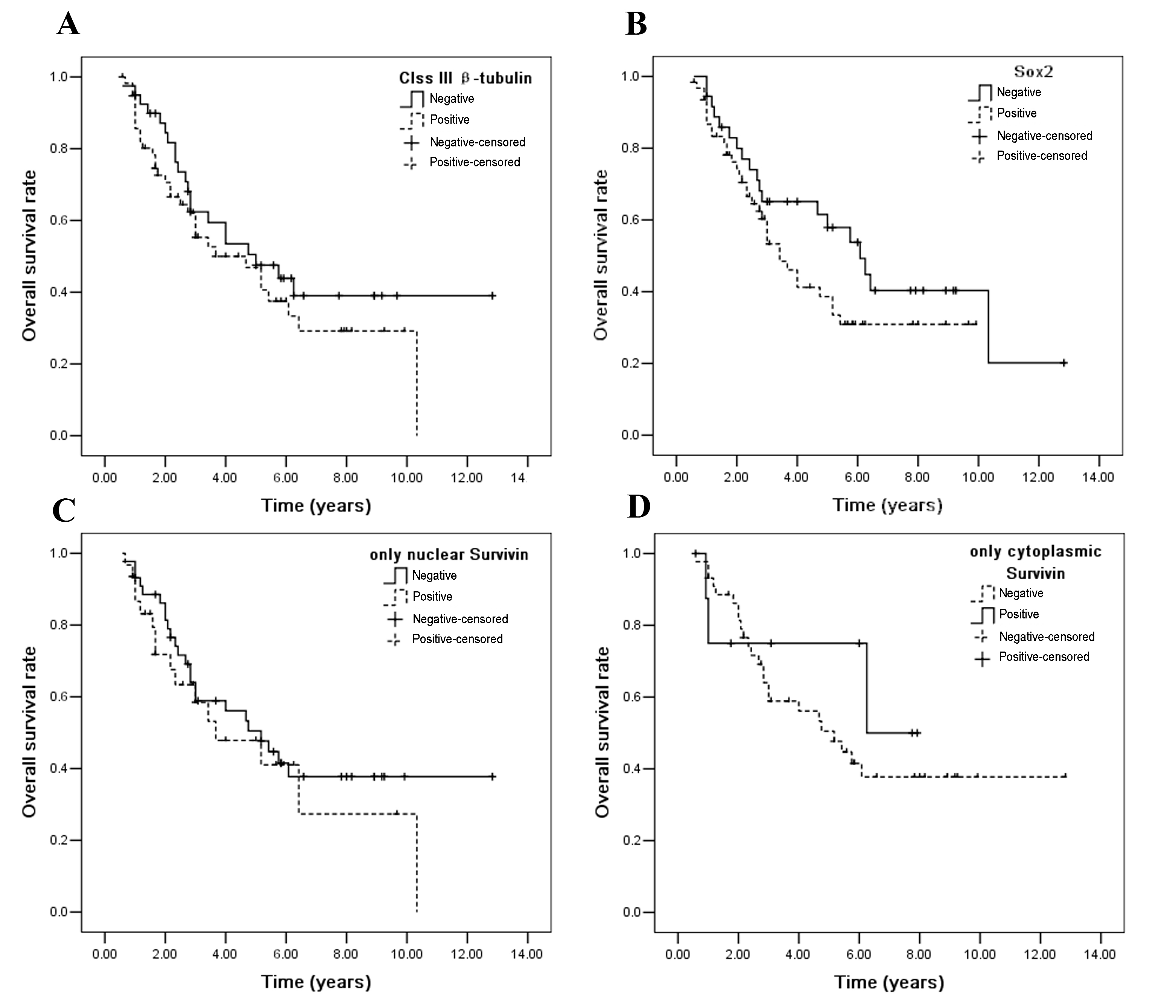

Supplement: Additional file 2: Figure S2. — Kaplan-Meier analysis for overall survival (OS) in 98 SOEC patients. There were no significant associations between expression of the three proteins and OS [p = 0.284, 0.138, 0.428, and 0.503 for Class III β-tubulin (A), Sox2 (B), only nuclear Survivin (C), and only cytoplasmic Survivin (D), respectively]. (JPEG 1010 kb) [file 12885_2015_1553_MOESM2_ESM.jpeg]
